# Does Pharmacological Adjustment Influence the Outcomes of In-Patient Multimodal Intensive Care? A Study in Patients with Moderately Advanced Parkinson’s Disease

**DOI:** 10.3390/jcm14165749

**Published:** 2025-08-14

**Authors:** Lyubov Rubin, Noureddin Elayan, Mara McCrossin, Cherie Roberts, Haque Shakil, Alessandro Di Rocco, Maria Felice Ghilardi

**Affiliations:** 1 Department of Neurology, Movement Disorders Division, Northwell Health, Donald and Barbara Zucker School of Medicine at Hofstra/Northwell, Long Island, NY 11542, USA; lrubin3@northwell.edu (L.R.); mmccrossin@northwell.edu (M.M.); croberts12@northwell.edu (C.R.); hshakil@northwell.edu (H.S.); adirocco1@northwell.edu (A.D.R.); 2 Department of Molecular, Cellular & Biomedical Sciences, CUNY School of Medicine, New York, NY 10031, USA

**Keywords:** Parkinson’s disease, rehabilitation, medication, inpatient treatment

## Abstract

**Background/Objectives**: We have previously shown that motor and non-motor symptoms of patients with Parkinson’s disease (PD) improved after a two-week in-patient multimodal intensive neurorehabilitation and care (iMINC). This program includes five hours/day for five days/week of multimodal neurorehabilitation and drug adjustments, taking advantage of extensive patient observation. In this study, we ascertained whether the improvements observed after iMINC similarly occurred in patients with and without drug adjustments. **Methods**: With a retrospective approach, the scores of UPDRS Total and Part III, Beck’s Depression Inventory (BDI), PDQ-39, Parkinson’s Disease Sleep Scale (PDSS), and Vocal Volume before and after two weeks of iMINC were compared in two groups of patients with moderate to advanced PD (H&Y Stage 3–4). In one group, drug adjustment was not necessary (PD no drug adjustment, PDnda, 38 patients), and another group underwent drug changes (PD with drug adjustment, PDda, 93 patients). Scores of all tests were compared using ANOVAs (within subject: before iMINC, after iMINC; between subject: PDda, PDnda). **Results**: Following iMINC, all outcome measures improved in both groups. **Conclusions**: Pharmacological adjustment is not the major factor that drives the improvement of motor and non-motor outcome scores following iMINC. These findings suggest that this comprehensive in-patient approach addresses most parkinsonian symptoms and that proper medication status may enhance the positive effects of iMINC.

## 1. Introduction

Patients with Parkinson’s disease (PD) experience not only motor symptoms, but also dysfunctions in the neuropsychiatric, sensory, and cognitive domains. As there is no cure for PD, symptomatic treatments other than dopaminergic or surgical therapies should be added and tailored to address the variety of symptoms. These additional treatments, which include physical therapy (PT), occupational therapy (OT), and speech therapy (ST), are usually performed in outpatient settings and may promote functional independence in patients over the short and long term [1,2,3].

In the last decade, in European centers, models of comprehensive care and multimodal intensive neurorehabilitation coupled with aerobic exercise have been developed in in-patient settings for a mean duration of three to four weeks. These in-patient treatments produce significant improvements in many motor and non-motor realms [2,4,5,6] and, in addition, they seem to delay disease progression [4]. An important component of this in-patient multimodal intensive neurorehabilitation and care (iMINC) is the adjustment of pharmacological treatment, taking advantage of patients’ observations in many contexts and during an extended period of hospitalization.

In the last few years, we have created, for the first time in the USA, an in-patient model of care for patients with PD at Glen Cove Hospital. The two-week iMINC treatment at Glen Cove includes PT, OT, ST, recreational and art therapy, neuropsychological care, dietary assessment as well as pharmacological adjustment when needed [7]. This occurs with the work of a multidisciplinary team consisting of physical medicine and rehabilitation specialists, neurologists with movement disorder expertise, nurses, pharmacists, and social workers. We have previously provided preliminary evidence that, at the end of the two-week iMINC treatment at Glen Cove, there was a significant improvement in most tests of motor and non-motor function. However, it remains unclear whether pharmacological adjustments play a major role in the observed improvements. Therefore, in the present retrospective study, we addressed this question by comparing the iMINC outcomes of two groups of patients with PD: one group undergoing pharmacological adjustment and the other without any pharmacological adjustment during the in-patient stay. We reasoned that if pharmacological adjustments were the main drivers of the observed improvements, the former group should show greater performance improvement than the latter.

## 2. Materials and Methods

### 2.1. Study Design

In this retrospective study, we reviewed the charts of 131 patients with PD who were admitted to the Parkinson’s Neurorehabilitation Unit at Glen Cove Hospital, whose admission was approved by Medicare or Insurance from January 2023 to November 2024. Patients were in Hoehn & Yahr Stages 3 to 4 and were admitted to the iMINC program based on the following criteria: disturbance of gait and mobility with a history of recent falls; lack of independence in daily life activities; swallowing and speech disorders; preservation of cognitive abilities; lack of improvement using outpatient care. For the present study, based on an automated protocol, patients were assigned to two groups: in the first group, patients underwent drug adjustments (PD with drug adjustment, PDda), and in the second group, patients maintained the same drug treatment during the entire in-patient stay (PD no drug adjustment, PDnda). The study was approved as exempt by the Institutional Review Board of the Feinstein Institutes for Medical Research—Northwell Health (IRB Protocol #25-0088-NSUH GC, 10 March 2025).

### 2.2. Intervention

**Figure 1:**
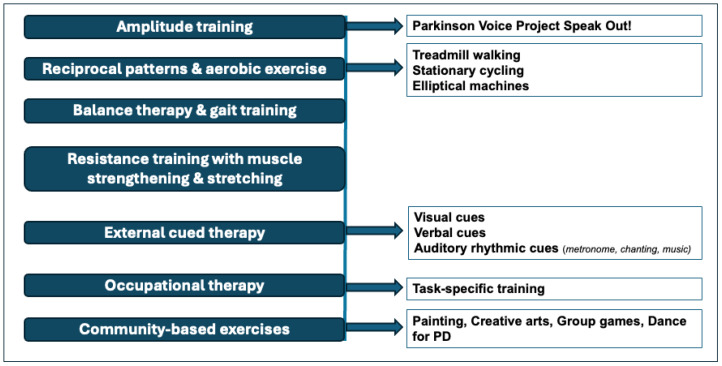
Daily activities during iMINC. The duration of each activity was adjusted based on each patient’s progress and needs. Swallowing and dysphagia therapy were incorporated into the protocol based on patient evaluation. For more details, see [7].

All patients underwent a two-week iMINC treatment that included PT, OT, and ST under the guidance of a multidisciplinary team, as detailed in a previous publication [7] and summarized in Figure 1. Briefly, this intensive treatment encompassed three hours of individualized PT, OT, and ST for five days a week, with an additional two hours of group therapy that included art and music therapies. The duration of each session, including warm-up and recovery periods, was approximately one hour for each discipline and adjusted based on patients’ progress and needs. As part of the team, the movement disorder specialists observed the patients and tailored pharmacotherapy throughout their in-patient stay.

### 2.3. Outcome Measures

Clinical and patient-reported outcome scales and tests evaluating motor and non-motor symptoms were administered and collected by trained personnel at both admission and discharge. Scales included Beck’s Depression Inventory (BDI), Parkinson’s Disease Sleep Scale (PDSS), Parkinson’s Disease Questionnaire—39 (PDQ-39), Timed Up and Go (TUG), Unified Parkinson’s Disease Rating Scale (UPDRS), and Vocal Volume. We also defined the parkinsonian medication regimen by computing the Levodopa Equivalent Dose (LED) at admission and discharge for all patients [8,9].

### 2.4. Data Analysis

We first analyzed possible differences between the PDda and PDnda groups in terms of age, length of hospital stay, LED at admission, and disease duration using Welch’s *t*-tests for unequal samples. Significance was set at 0.05 for *p*-value and greater than 2.46 for Vovk-Sellke maximum *p*-ratio (VS-MPR) [10]. VS-MPR is based on *p*-values and represents the lower bound of the Bayes Factor, favoring H0 over H1 for a wide range of different prior distributions, thus assuring that the absolute probability of the model can provide an acceptable explanation of the results. Finally, when appropriate, effect size was determined with Cohen’s d values and 95% confidence intervals (CI). For all outcome measures, we then analyzed changes possibly related to iMINC with ANOVAs, using time (pre- and post-iMINC) as a within-subject factor and drug adjustment status (PDda and PDnda) as a between-subject factor. We used post-hoc tests when appropriate. We set the threshold of *p*-value significance at 0.05 and for VS-MPR, which measures the strength of evidence against the null hypothesis, at values higher than 2.46. To verify effect size, we computed partial eta-squared (η^2^ *p*), i.e., the % of the variance in the dependent variable attributable to a particular independent variable. We also performed Bayesian correlations with Pearson rho and BF_10_ for each motor and non-motor score improvement with length of stay, age, LED, and disease duration. Further statistical analyses were based on comparisons between patients in the PDna group, those in whom the LED increased (PDda+), and those in whom the LED decreased (PDda-) during iMINC. Methods and results of these analyses are reported in the Supplementary Material. We used JASP (version 0.19) for all statistical analyses.

## 3. Results

A total of 131 subjects with PD underwent the iMINC program at Glen Cove Hospital. During the in-patient stay, drug adjustments were made in 93 out of 131 patients (PDda group), with LED dosage decreasing in 40 of them and increasing in 53 of them. The remaining 38 patients did not have any pharmacological changes and were allocated to the PDnda group. The characteristics of the two groups are presented in Table 1. Briefly, there were more men than women in both groups (ratios of 2:1 and 1.5:1 in the PDda and PDnda groups, respectively). Moreover, patients in the PDda group were, on average, slightly younger but with longer disease duration and stayed in the hospital for an average of almost a day longer. Nevertheless, because of the 95% CI values and the fact that VS-MPR values were very close to 2, all these results should be interpreted as weak evidence against the null hypothesis. The PDda group also had higher values of LED at both admission and discharge compared to the PDnda group.

We then ascertained possible group differences for the outcomes before and after iMINC. The results of mixed-model ANOVAs are reported in Table 2, together with the mean and SD of the outcome measures for the two groups before and after iMINC. Briefly, we found a group difference in the UPDRS total score at baseline, in that the PDda group had higher scores, and thus more severe symptoms, than the PDnda group, as attested by a significant group effect (see UPDRS total in Table 2) and by a significant post-hoc test comparing PDda and PDnda at baseline (mean difference = 11.855, t = 2.522, *p* = 0.026, Cohen’s d = 0.504, 95% CI −0.039 1.048). Such group differences were not present after iMINC (mean difference = 6.019, t = 1.362, *p* = 0.176, Cohen’s d = 0.256, 95% CI −0.250 0.762). Nevertheless, the effect of iMINC was similar in the two groups, as both achieved a similar decrease of the total UPDRS score (see Table 2). For the motor scores of UPDRS III, we found that the group difference showed only a trend towards significance (see Table 2), with the PDda group having somewhat greater scores than the PDnda group. In any case, both groups similarly improved after iMINC, with an average score reduction of more than 10 points (See Table 2).

The analysis of PDQ 39 showed some group differences that, however, were not confirmed by post-hoc tests (PDda vs. PDnda pre-iMINC: mean difference = 6.142, t = 1.953, *p* = 0.31, Cohen’s d = 0.37, 95% CI −0.146 0.904; post-iMINC: mean difference = 6.704, t = 2.017, *p* = 0.275, Cohen’s d = 0.414, 95% CI −0.141 0.969). It should be noted that all the described group differences had rather small effect sizes, with η^2^ *p* values always less than 0.04 (see Table 2). Depression (BDI), sleep quality (PDSS), and voice volume improved after iMINC, without differences between the two groups (See Table 2). None of the motor and non-motor score improvements significantly correlated with length of stay, age, LED, or disease duration (Pearson’s rho from −0.094 to 0.071; BF_10_ from 0.111 to 0.192). Further analyses within the PDda group comparing patients with increased LED and those with decreased LEDs did not reveal any differences between the two PDda subgroups across all outcome measures (all *p* > 0.1).

In summary, the results of the present analyses suggest that, after iMINC, motor scores, as well as quality of life, mood, sleep, and vocal performance, improved independently of drug adjustment and group characteristics at baseline.

## 4. Discussion

The results of our analyses demonstrated that, after the two-week iMINC at Glen Cove Hospital, patients in both PDda and PDnda groups showed improvements in motor and non-motor functions. The sizes of the improvements were similar in the two groups, without significant differences. Moreover, in both groups, the score changes in the tests we used were not only statistically significant but also clinically meaningful, in line with our previous report [7].

We first discuss the possible differences between the two patient groups at baseline. Interestingly, the PDda group was slightly younger, with a relatively longer disease duration than the PDnda group. Indeed, the PDda group had worse scores of UPDRS Total, PDQ-39, and UPDRS III at baseline. PDQ39 represents a general feeling about the quality of patients’ lives, while UPDRS total score is a composite measure of the interaction and effect of the disease on activities of daily living, sleep, medication side effects, such as levodopa-induced dyskinesias, and motor function. These characteristics are in line with situations guiding the need for therapy adjustment, as the frequency of motor complications generally increases with longer exposure to dopaminergic therapy and at a younger onset of disease with more advanced stages of the disease. Among the PDda group, there was no difference between the patients in whom the LED was decreased (43%) and those in whom the LED was increased (57%) (see Supplementary Tables S1 and S2). In any event, the two-week period of in-patient stay provides a great opportunity to review and adjust medications, as it allows for continuous and direct examination of the patient in various contexts, activities, and situations. Moreover, the constant observation of many people with different expertise and points of view, including the patients themselves, the on-site therapists, and the family members, allows the movement disorder specialist to change and verify the effects of the medication adjustment. Thus, iMINC represents a unique opportunity to overcome the limitations of the outpatient clinical setting, where patients are usually assessed at their best and over a very short period of time.

Nevertheless, some limitations are ascribed to factors that could mask the potential differences between the PDda and PDnda groups. First, the sample sizes were unbalanced, with PDda constituting approximately 70% of the patients. Second, during iMINC, some patients in the PDda group had increased LED (PDda+) and others had decreased LED (PDda). We addressed these problems with analyses comparing the three groups (PDna, PDda+, and PDda-) that are reported in the Supplementary Material. Remarkably, while the three groups did not differ in terms of age, the PDda group had a longer PD duration (from 5 to 6 years) and were on much higher levels of LED compared to the other two groups (Supplementary Tables S1 and S2). Despite differences in baseline characteristics, both analytical approaches clearly showed that all groups similarly benefited from iMINC treatment, suggesting that this comprehensive in-patient program can improve most of the Parkinsonian symptoms (see Table 2 and Supplementary Table S3). In addition, we found that higher baseline UPDRS scores, longer disease duration, and higher LED did not predict the size of motor and non-motor scores. In any event, it is plausible that, at least in part, a proper medication status may contribute to the positive outcomes of iMINC by working on at least two aspects. Indeed, titration of dopaminergic drugs in patients with PD has direct effects on symptoms: drug increases may reduce some motor problems, such as OFF periods, and drug decreases may lessen dyskinesias. In both situations, the quality of life should improve, thus providing a better setting for the activities involved in the iMINC. On the other side, levels of dopaminergic therapy levels influence plasticity processes involved in long-term potentiation and their behavioral expression. These plasticity processes, which are important for inducing and maintaining the effects of exercise, are impaired in PD [11,12,13]. In addition, the fact that levodopa acutely restores these processes in non-dyskinetic but not in dyskinetic patients [11] can also explain why both increases and decreases in dopaminergic drugs produce positive effects. As discussed in a previous paper, exercise likely remains the main driver of improved plasticity [7].

Finally, and most importantly, in both groups, the changes in motor and non-motor test scores following iMINC were not only statistically significant, but on average, they were also clinically meaningful, as we previously reported in a different patient cohort [7]. For example, UPDRS total scores improved by an average of 28 and 22 points in the PDda and PDnda groups, respectively, with an average decrease of 18 points considered a large clinically important difference [14]. Similarly, the motor UPDRS III scores decreased by an average of more than 10 points in both groups, a change considered a large clinically important difference [14]. Comparable results in the range of clinically meaningful difference (CMD) were found for other measures: indeed, BDI improved by more than 4 points (or 30% decrease, minimum CMD: 2 points [15]); PDQ39 total score by more than 4.3 points (minimum CMD: 1.6 points [16]); and PDSS by 17 points (minimum CDM: 3.44 points [17]). These results suggest that participation in intensive workouts in a protected environment may add psychological benefits, thus increasing the sense of achievement, further activating the reward system, and enhancing plasticity processes [18,19,20,21]. Finally, as suggested by Choi et al. [22], multimodal rehabilitation in patients with PD can also reduce the caregiver burden. Altogether, our results suggest that iMINC induces a clinically meaningful improvement in motor and non-motor spectra and imply a possible, although temporary, slowdown of the disease progression. As suggested by some European studies that employ programs of longer duration (up to four weeks) [4,5,6], repeating iMINC over the years may delay disease progression. In our case, iMINC is a relatively young program in the US and is currently paid for by Medicare for a two-week duration. It would be important to compare the long-term effects of our two-week program with those of four-week programs to understand whether longer programs would produce more and longer-lasting effects and ultimately decrease the social and economic costs of PD. Only follow-up studies can answer this question.

This study has some limitations that were, in part, addressed in the previous paragraphs, such as the baseline differences between the groups. Among other limitations, which include the relatively small number of patients per group and the lack of follow-up, the retrospective nature of the study does not allow us to assess the duration of the benefits. However, the retrospective approach is significantly cheaper, easier to carry out, and can serve as a guide for extensive studies. Indeed, the results of retrospective studies can be used to identify patterns and measure effect sizes to plan prospective studies. In addition, retrospective studies may better mimic real-life scenarios, as controlled environments may mask some aspects. Despite these limitations, the results of this study further confirm the viability of iMINC as a comprehensive treatment model for patients with PD, with medication adjustment possibly playing a role not only in symptom management but also in plasticity enhancement. Future studies should address the limitations described above, including the reproducibility of the present results, also within outpatient settings, and a longer follow-up with several testing points.

In summary, iMINC and similar care models offer room for evolving and elevating the level of care in every aspect of PD. Efforts such as this study are worthwhile because they allow to explore novel approaches to treat patients, enhance their quality of life, and address the dire need for cost-effective interventions. In fact, it is estimated that by 2037, the total economic burden of PD will reach at least 79 billion dollars, a figure surpassing previously estimated values [23]. It is important to note that, in 2017, hospitalization alone cost 7 billion dollars, representing 28.4% of direct medical costs [23]. Integrated models, such as iMINC, could reduce the number of hospitalizations and falls, positively impacting health care costs and the long-term economic burden of PD.

## Figures and Tables

**Table 1:** Characteristics of patient groups. Mean and Standard deviation (SD) are reported for age, in-patient stay, PD duration, Levodopa Equivalent Dose (LED) at admission, PDda: patients with PD undergoing drug adjustment, PDnda: patients with PD without drug adjustment, n: number of subjects, m: male, f: female, VS-MPR: Vovk Sellke Maximum p-Ratio, 95% CI: 95% Confidence Interval. Statistically significant results are in bold.

	PDda (n = 93)	PDnda (n = 38)	Welch’s Test Results of Group Comparison
Sex (m:f)	62:31	23:15	-
Age (yrs)	74.2 ± 8.5	77.3 ± 7.6	t = 2.03, ***p* = 0.046**, VS-MPR = 2.60, Cohen d’s = −0.38, 95% CI −0.761 0.003
In-patient stay (days)	14.6 ± 2.4	13.9 ± 2.2	t = 2.91, ***p* = 0.034**, VS-MPR = 3.32, Cohen d’s = 0.384, 95% CI 0.778 0.014
PD duration (yrs)	12.0 ± 8.6	8.7 ± 7.9	t = 2.09, ***p* = 0.04**, VS-MPR = 2.86, Cohen d’s = 0.4, 95% CI 0.012 0.795
LED at admission	820 ± 460	657 ± 354	t = 2.18, ***p* = 0.032**, VS-MPR = 3.36, Cohen d’s = 0.397, 95% CI 0.778 0.014
LED at discharge	875 ± 441	657 ± 354	t = 2.97, ***p* = 0.004**, VS-MPR = 17.17, Cohen d’s = 0.545, 95% CI 0.930 0.158

**Table 2:** Means and SDs of the outcome measures for PDda and PDnda groups are reported together with the results of mixed model ANOVAs. Effects of iMINC within-subject effects (time: Pre vs. Post iMINC) and between-subject effects (group: PDda vs. PDnda). Significant results are reported in bold. PDda: patients with PD undergoing drug adjustment; PDnda: patients with PD without drug adjustment; N: number of subjects; *p*: *p*-value; VS-MPR: Vovk Sellke Maximum *p*-Ratio. For each measure, we also reported the number of subjects in each group who underwent pre- and post-iMINC evaluations.

Test	PDda (Mean ± SD)	PDnda (Mean ± SD)	iMINC	Group	iMINC × Group
*UPDRS Total* Pre Post	N = 88 110.2 ± 22.8 81.9 ± 21.4	N = 38 98.3 ± 27.3 75.9 ± 25.8	**F = 127.6 *p* < 0.001** VSMPR = 9.12 × 10^+17^ η^2^ *p* = 0.507	**F = 5.0 *p* = 0.026** VS-MPR = 3.84 η^2^ *p* = 0.039	F = 1.7 *p* = 0.19 VS-MPR = 1.15 η^2^ *p* = 0.014
*UPDRS III* Pre Post	N = 88 56.1 ± 14.2 43.9 ± 12.3	N = 38 51.5 ± 13.4 40.4 ± 13.6	**F = 85.9 *p* < 0.001** VSMPR = 1.46 × 10^+13^ η^2^ *p* = 0.409	F = 3.2 *p* = 0.076 VS-MPR = 1.88 η^2^ *p* = 0.025	F = 0.19 *p* = 0.66 VS-MPR = 1.00 η^2^ *p* = 0.002
*BDI* Pre Post	N = 83 14.3 ± 7.7 9.8 ± 6.9	N = 33 14.4 ± 9.5 10.8 ± 7.6	**F = 65.9 *p* < 0.001** VSMPR = 2.14 × 10^+10^ η^2^ *p* = 0.366	F = 0.14 *p* = 0.70 VS-MPR = 1.00 η^2^ *p* = 0.001	F = 0.92 *p* = 0.33 VS-MPR = 1.00 η^2^ *p* = 0.008
*PDQ-39* Pre Post	N = 88 47.3 ± 14.6 43.2 ± 16.9	N = 35 41.2 ± 18.4 36.5 ± 15.9	**F = 17.7 *p* < 0.001** VSMPR = 767.28 η^2^ *p* = 0.128	**F = 4.4 *p* = 0.038** VS-MPR = 2.96 η^2^ *p* = 0.035	F = 0.07 *p* = 0.78 VS-MPR = 1.00 η^2^ *p* = 5.95 × 10^−4^
*PDSS* Pre Post	N = 85 91.9 ± 25 109.8 ± 20.1	N = 33 97.2 ± 22.6 112.8 ± 22.9	**F = 66.2 *p* < 0.001** VS-MPR = 5.95 × 10^−4^ η^2^ *p* = 0.363	F = 0.97 *p* = 0.32 VS-MPR = 1.01 η^2^ *p* = 0.008	F = 0.30 *p* = 0.58 VS-MPR = 1.00 η^2^ *p* = 0.003
*Vocal Volume* Pre Post	N = 92 50.9 ± 5.0 57.8 ± 6.0	N = 36 51.9 ± 5.2 59.1 ± 5.5	**F = 277.5 *p* < 0.001** VSMPR = 4.01 × 10^+30^ η^2^ *p* = 0.688	F = 1.25 *p* = 0.26 VS-MPR = 1.04 η^2^ *p* = 0.010	F = 0.21 *p* = 0.64 VS-MPR = 1.00 η^2^ *p* = 0.002

## Data Availability

De-identified data will be provided upon request to the corresponding authors and approval of their institutions.

## Supplementary Materials

The following supporting information can be downloaded at: https://www.mdpi.com/article/10.3390/jcm14165749/s1, Table S1: Characteristics of patient groups and the results of ANOVA determining possible group effects; Table S2: Results of post hoc test for: age, in-patient stay, PD duration, Levodopa Equivalent Dose (LED) at admission and discharge; Table S3: Means and SDs of the outcome measures for PDda+, PDda- and PDnda groups (first three columns) are reported together with the results of mixed model ANOVAs (last three columns).
