# Supplementary material for: Does Pharmacological Adjustment Influence the Outcomes of In-Patient Multimodal Intensive Care? A Study in Patients with Moderately Advanced Parkinson’s Disease
Source: J Clin Med. 2025 Aug 14;14(16):5749. doi: 10.3390/jcm14165749 (PMC12386698; doi:10.3390/jcm14165749)
Supplement: Supplementary file 1 [file jcm-14-05749-s001.zip › jcm-3741548-supplementary.pdf]

Supplemental material

Supplemental Data Analysis

To verify whether the direction of drug adjustment (increase vs. decrease) influenced the results and to ascertain possible biases due to unequal groups' sample size, we divide the PD cohort of the iMINC program in three groups: patients that underwent LED increase during inpatient treatment (PDda+), patients that underwent LED decrease (PDda-), and patients with PD without drug adjustment (PDnda). We first verified possible group differences in terms of age, length of hospital stay, LED at admission, and disease duration with factorial ANOVA followed, when appropriate, by post hoc with Bonferroni correction. P-values were significant when lower than 0.05 and VS-MPR (a measure of evidence against the null hypothesis) more than 2.46. Effect size (Cohen's d values, 95%) and confidence intervals (CI) were also computed. We then performed ANOVA with time (pre- and post-iMINC) as within-subject factor and drug adjustment status (PDnda, PDda+, PDda-) as between-subject factor followed by appropriate post hoc tests. Effect size was ascertained with η^2 p. Results were significant when p-value was lower than 0.05 and VS-MPR more than 2.46. Analyses were conducted with JASP (version 0.19).

Supplemental Results

During iMINC, LED dosage was decreased in 40 patients (PDda- group) and increased in 53 (PDda+ group), while 38 patients did not have any change (PDnda group). Supplementary Table 1 reports the characteristics of the three groups and the results of their comparison with ANOVAs. In supplementary Table 2, we report the results of post hoc tests for the significant comparisons. Briefly, while there was no difference in terms of age, ANOVA and post hoc tests suggest that the PDda- group had longer PD duration, had a slightly longer stay in the hospital and were on higher levels of LED. At discharge LED dosage was greater in the PDda+ group.

Supplementary Table S1. Characteristics of patient groups and the results of ANOVA determining possible group effects. Mean ± Standard deviation are reported for: age, in-patient stay, PD duration, Levodopa Equivalent Dose (LED) at admission and discharge, PDda+: patients with LED increase, PDda-: patients with LED decrease, PDnda: patients without drug adjustment, n: number of subjects, VS-MPR: Vovk Sellke Maximum p-Ratio.

	PDda+ (n=53)	PDda- (n=40)	PDnda (n=38)	ANOVA Group effect
Age (yrs)	74.2 ±9.0	74.3 ±8.3	77.3 ±7.6	F=1.84, p=0.163, VS-MPR=1.24, η^2 p=0.028
In-patient stay (days)	14.4±2.4	15.6±3.7	13.9±2.2	**F=3.79, p=0.025,** VS-MPR=3.96, η^2 p=0.056
PD duration (yrs)	9.7 ± 7.1	15.2 ± 9.6	8.7 ± 7.9	**F=7.6, p=0.001,** VS-MPR=47.6, η^2 p=0.039
LED at admission	645 ± 365	1051 ± 473	657 ± 354	**F=14.1, p<0.001,** VS-MPR=9936, η^2 p=0.18
LED at discharge	950 ± 513	775 ± 301	657 ± 354	**F=5.8 p=0.004,** VS-MPR=17.2, η^2 p=0.083

Supplementary Table S2. Results of post hoc test for: age, in-patient stay, PD duration, Levodopa Equivalent Dose (LED) at admission and discharge, PDda+: patients with LED increase, PDda-: patients with LED decrease, PDnda: patients without drug adjustment. Only the significant comparisons (p<0.05 with Bonferroni correction for multiple comparisons) are fully reported.

Comparison	Mean difference	t value	p value	Cohen's d	95% CI
Inpatient stay					
PDda- vs PDda+	1.17				
PDda- vs PDnda	1.68	3.43	0.026	0.60	0.182 3.175
PDda+ vs PDnda	0.51				
PD duration					
PDda- vs PDda+	5.48	4.87	0.006	0.668	1.359 1.551
PDda- vs PDnda	6.51	4.37	0.002	0.794	2.012 11.00
PDda+ vs PDnda	1.03				
LED at admission					
PDda- vs PDda+	406	4.87	0.001	1.02	208 604
PDda- vs PDnda	394	4.37	0.001	0.99	0.489 1.551
PDda+ vs PDnda	-12				
LED at discharge					
PDda- vs PDda+	-175				
PDda- vs PDnda	118				
PDda+ vs PDnda	293	3.34	0.003	0.719	0.184 1.237

We then analyzed the effects of iMINC in the three groups. In Supplementary Table 3, we report the results of ANOVAs together with the mean and SD of all the outcome measures for the three groups before and after iMINC. Briefly, we found a significant improvement of all the measures in all the three groups without any group differences or group X iMINC effect, suggesting that iMINC produced a similar benefit across the groups. Indeed, post-hoc tests showed significant pre- post-iMINC differences in all the groups for all the measures with the exception of PDQ39 in the PDda- group that showed only a trend (p=0.10) toward improvement.

Supplementary Table S3. Means and SDs of the outcome measures for PDda+, PDda- and PDnda groups (first three columns) are reported together with the results of mixed model ANOVAs (last three columns). For each measure, we also report the number of subjects in each group that had pre- and post-iMINC evaluation. Significant results are reported in bold. PDda+: patients with PD and LED increase, PDda-: patients with PD and LED decrease, PDnda: patients with PD without drug adjustment, N: number of subjects, p: p-value; VS-MPR: Vovk Sellke Maximum p-Ratio.

Test	PDda+ (mean±SD)	PDda- (mean±SD)	PDnda (mean±SD)	iMINC	Group	iMINC x Group
UPDRSTot.	N= 51	N= 37	N= 38	**F=163.4 p<0.001**	F=2.6 p=0.076	F=1.39 p=0.25
Pre	108.2±23.6	112.9±21.5	98.3±27.3	VSMPR=2.7×10^{+21}	VS-MPR=1.88	VS-MPR=1.057
Post	82.1±21	81.7±22.1	75.9±25.8	η^2 p=0.571	η^2 p=0.041	η^2 p=0.022
UPDRSIII	N= 51	N=37	N= 38	**F=105.6 p<0.001**	F=1.6 p= 0.21	F=0.58 p= 0.56
Pre	55.7±14.8	56.7±13.5	51.5 ± 13.4	VSMPR=3.1×10^{+15}	VS-MPR=1.13	VS-MPR=1.00
Post	44.6±11.1	42.9±13.8	40.4 ± 13.6	η^2 p= 0.462	η^2 p=0.025	η^2 p=0.009
BDI	N= 48	N= 35	N= 33	**F=91.5 p<0.001**	F=0.58 p=0.56	F=2.24 p=0.111
Pre	14.5±8.4	13.9±6.7	14.4±9.5	VSMPR=3.3×10^{+13}	VS-MPR=1.00	VS-MPR=1.51
Post	10.9±8.2	8.2±4.2	10.8±7.6	η^2 p=0.45	η^2 p=0.01	η^2 p=0.038
PDQ-39	N= 49	N= 39	N= 35	**F=19.9 p<0.001**	F=2.5 p=0.085	F=0.249 p=0.78
Pre	46.5±15.7	48.4±13.1	41.2±18.4	VSMPR=1811.8	VS-MPR=1.76	VS-MPR=1.00
Post	41.7±17.4	45±16.2	36.5±15.9	η^2 p=0.142	η^2 p=0.04	η^2 p=0.004
PDSS	N=49	N=36	N= 33	**F=82.4 p<0.001**	F=1.0 p=0.344	F=0.17 p=0.85
Pre	93.8±27.1	89.4±21.9	97.2±22.6	VS-MPR=3.0×10^{+12}	VS-MPR=1.00	VS-MPR=1.00
Post	112±18.9	106.7±21.4	112.8±22.9	η^2 p=0.417	η^2 p=0.018	η^2 p=0.003
Vocal Vol.	N= 53	N= 39	N= 36	**F=326.6 p<0.001**	F=0.7 p=0.518	F=0.15 p=0.86
Pre	51.2±4.6	50.7±5.6	51.9±5.2	VSMPR=3.9×10^{+30}	VS-MPR=1.00	VS-MPR=1.00
Post	57.9±5.1	57.7±7.2	59.1±5.5	η^2 p=0.723	η^2 p=0.010	η^2 p=0.002
